# Cigarette smoking history, e-cigarette use, and Long COVID: a population-based analysis

**DOI:** 10.1007/s11739-026-04512-w

**Published:** 2026-08-28

**Authors:** Yusuff Adebayo Adebisi, Anas Ali Alhur

**Affiliations:** 1 College of Social Sciences, University of Glasgow, 40 Bute Gardens, Glasgow, G12 8RT UK; 2 Department of Clinical and Experimental Medicine, University of Catania, Catania, Italy; 3 Department of Health Informatics, College of Public Health and Health Informatics, University of Hail, Hail, Saudi Arabia

**Keywords:** Long COVID, E-cigarettes, Vaping, Cigarette smoking, Dual use, Scottish Health Survey, Epidemiology

## Abstract

Long COVID imposes a substantial public health burden following the SARS-CoV-2 pandemic, yet the contribution of electronic cigarette (e-cigarette) use, alone or alongside cigarette smoking, remains inadequately characterized in population-representative data. We conducted a cross-sectional analysis of 9274 adults aged 16 years and older from the pooled 2023–2024 Scottish Health Survey, a nationally representative household survey. Cigarette smoking and e-cigarette use were classified into seven mutually exclusive categories, and the outcome was survey-defined self-reported Long COVID, based on symptoms persisting for more than four weeks after first having COVID-19. Survey-weighted logistic regression was used to estimate crude and adjusted odds ratios, with adjustment for survey year, age group, sex, area deprivation, ethnicity, alcohol use, and educational attainment. Overall, 7.4% of adults reported Long COVID. Compared with never cigarette/e-cigarette users, adjusted odds were higher among former cigarette smokers currently using neither product (adjusted odds ratio 1.40, 95% confidence interval 1.12–1.76) and among former smokers currently using e-cigarettes exclusively (1.56, 1.09–2.24). No statistically detectable associations were observed among former e-cigarette users who had never smoked, current e-cigarette users who had never smoked, exclusive current cigarette smokers, or dual users. The two elevated associations persisted after additional adjustment for asthma, chronic obstructive pulmonary disease, diabetes, and cardiovascular disease. The observed pattern may reflect previous cigarette exposure, health-related smoking cessation or switching, reverse causation, and residual selection or confounding; the cross-sectional design does not permit an independent contribution of e-cigarette use to be determined.

**Supplementary Information:**

The online version contains supplementary material available at https://doi.org/10.1007/s11739-026-04512-w.

## Introduction

The severe acute respiratory syndrome coronavirus 2 (SARS-CoV-2) pandemic has left a substantial burden of persistent symptoms among people who continue to experience fatigue, breathlessness, cognitive problems, and other health complaints weeks or months after acute infection. This condition, commonly referred to as Long COVID or post-COVID-19 condition, remains an important public health concern because of its implications for quality of life, work capacity, and healthcare use [1–3]. In the United Kingdom, national estimates have shown that persistent post-COVID symptoms have affected large numbers of adults, underscoring the continuing relevance of Long COVID beyond the acute phase of the pandemic [4]. For example, around 2 million people living in private households in the United Kingdom, representing approximately 3% of the population, were estimated to be experiencing self-reported Long COVID in March 2023.

Identifying behavioral factors associated with Long COVID remains an important research priority. Tobacco smoking is an established risk factor for respiratory disease and has been linked consistently to worse acute COVID-19 outcomes [5]. Its relationship with Long COVID, however, is less straightforward. While some studies have reported higher odds of persistent post-COVID symptoms among current or former smokers, others have found that the association attenuates after adjustment for demographic, socioeconomic, or health-related factors, suggesting that pre-existing morbidity may account for part of the observed excess risk [6]. A recent UK longitudinal analysis, for example, found no statistically significant independent association between pre-pandemic smoking and Long COVID after adjustment despite a borderline crude association [7].

Interpretation is further complicated by the broader debate around the so-called smoker’s paradox in COVID-19 research. During the pandemic, some observational studies appeared to show lower-than-expected rates of SARS-CoV-2 infection or adverse outcomes among current smokers [8–10], prompting speculation about a possible protective effect of nicotine [11–13]. Subsequent reviews have argued that this pattern is unlikely to reflect a simple biological benefit and may instead arise from selection bias, collider bias, under-ascertainment, differential survival, or other methodological distortions [14]. The nicotine hypothesis therefore remains contested rather than established.

The role of electronic cigarettes is also uncertain. On the one hand, e-cigarette aerosol generally contains fewer harmful chemicals than cigarette smoke, and e-cigarettes are widely regarded as less harmful than combustible tobacco, although not risk-free [15]. On the other hand, vaping still exposes the respiratory tract to nicotine, particulates, carbonyls, metals, and other constituents that may affect airway inflammation, epithelial integrity, and host defense [16]. This has led to competing expectations: if lower toxicant exposure relative to smoking is what matters most, e-cigarette users might be expected to show lower risk than smokers; if ongoing aerosol exposure or nicotine-related mechanisms are relevant, vaping could still be associated with persistent post-COVID symptoms in some users.

Direct evidence on e-cigarettes and Long COVID remains sparse. The clearest population-based study to date, based on the 2022 Behavioral Risk Factor Surveillance System, reported no independent association between current e-cigarette use and post-acute COVID-19 syndrome after adjustment [17]. However, studies in this area often treat e-cigarette users as a single category, despite substantial heterogeneity in prior smoking history. Current e-cigarette users may include former smokers who switched from cigarettes, dual users who continue to smoke, and adults who have never smoked cigarettes at all. These groups are unlikely to have the same baseline respiratory risk, cumulative tobacco exposure, or motivations for product use, and collapsing them together may obscure important differences in relation to Long COVID.

Against this background, the key unresolved question is not simply whether current e-cigarette use is associated with Long COVID, but whether any observed burden is concentrated in specific subgroups, particularly former smokers who now use e-cigarettes, rather than among never-smoking e-cigarette users, former e-cigarette users who never smoked, or current smokers more generally. This distinction matters because it speaks directly to whether the pattern is more consistent with prior combustible tobacco exposure, current vaping, behavioral responses to illness, or some combination of these factors. We therefore examined the association between cigarette and e-cigarette use patterns and Long COVID among adults in Scotland, distinguishing current and former e-cigarette use by cigarette-smoking history and separating exclusive cigarette smoking from dual use.

## Materials and methods

### Study design, data source, and study population

This study used a cross-sectional design based on pooled data from the 2023 and 2024 waves of the Scottish Health Survey (SHeS), an annual, nationally representative household survey of individuals’ residents in private households in Scotland [18]. The SHeS employs a stratified, clustered probability sample and collects detailed information on health status, health behaviors, and sociodemographic characteristics through face-to-face interviews conducted by trained interviewers [18].

The pooled 2023–2024 dataset initially comprised 13,710 respondents. Restriction to adults aged 16 years and older reduced the sample to 9538. We subsequently excluded 96 respondents with missing information on current e-cigarette/cigarette use pattern and 168 with missing or non-substantive responses on Long COVID status, yielding a final analytic sample of 9274 participants. All analyses incorporated the complex survey design by applying the pooled 2023–2024 interview weight together with the primary sampling unit and stratum identifiers supplied with the dataset, ensuring that estimates are representative of the adult population of Scotland.

### Outcome measure

The outcome of interest was survey-defined self-reported Long COVID. Respondents were classified as having Long COVID if they reported that they were still experiencing symptoms more than four weeks after first having COVID-19. Responses were coded as a binary indicator for the presence or absence of self-reported Long COVID. In the final analytic sample, 684 respondents reported Long COVID and 8590 did not. This operational definition reflected the wording available in the Scottish Health Survey and was not equivalent to a clinical diagnosis.

### Exposure measure

The principal exposure was cigarette and e-cigarette use pattern, operationalized as a seven-level mutually exclusive variable: (i) never cigarette/e-cigarette users, defined as respondents who had never smoked cigarettes and had never used e-cigarettes; (ii) former cigarette smokers currently using neither product, regardless of whether they had previously used e-cigarettes; (iii) former e-cigarette users who had never smoked cigarettes and currently used neither product; (iv) exclusive e-cigarette users who were former smokers, defined as current e-cigarette users who had previously smoked cigarettes but did not currently smoke; (v) exclusive e-cigarette users who had never smoked cigarettes; (vi) exclusive current cigarette smokers who did not currently use e-cigarettes; and (vii) dual users who currently both smoked cigarettes and used e-cigarettes. Within the analytic sample, these groups comprised 4900, 2409, 152, 504, 119, 949, and 241 respondents, respectively. The survey items used in this analysis identified cigarette smoking and e-cigarette or vaping-device use but did not separately identify heated-tobacco products; heated-tobacco use could therefore not be analyzed as a distinct category.

### Covariates

Covariates were selected a priori based on their plausibility as confounders of the association between cigarette and e-cigarette use pattern and self-reported Long COVID [6, 7, 19]. These included survey year (2023 or 2024); age group (16–24, 25–34, 35–44, 45–54, 55–64, 65–74, and 75 years or older); sex (male, female, prefer not to say); area deprivation, measured using quintiles of the Scottish Index of Multiple Deprivation (SIMD) from most to least deprived; ethnicity (White Scottish, Other White, minority ethnic); alcohol use (never drank alcohol, ex-drinker, drinks within guidelines, drinks above guidelines, missing/non-substantive), with drinking guidelines defined according to the UK Chief Medical Officers’ low-risk drinking threshold of no more than 14 units per week [20]; and educational attainment (degree or higher, HNC/HND or equivalent, Higher grade or equivalent, Standard grade or other school-level qualifications, no qualifications). A sensitivity model additionally included doctor-diagnosed asthma, chronic obstructive pulmonary disease (COPD), diabetes excluding gestational diabetes, and cardiovascular disease excluding diabetes and hypertension.

### Statistical analysis

Descriptive statistics was computed as unweighted counts with survey-weighted column percentages. Differences in the distribution of characteristics by Long COVID status were evaluated using design-based tests that accounted for weighting, clustering, and stratification. Associations between cigarette and e-cigarette use pattern and self-reported Long COVID were examined using survey-weighted binary logistic regression. We first fitted a crude model including product-use pattern only and then a multivariable model adjusted for age group, sex, area deprivation, ethnicity, alcohol use, educational attainment, and survey year. Results are reported as odds ratios (ORs) with 95% confidence intervals (CIs) and two-sided p-values. The overall contribution of the product-use variable was assessed using an adjusted Wald test, and adjusted predicted probabilities were obtained using marginal standardization.

Two sensitivity analyses were conducted. First, former cigarette smokers currently using neither product were subdivided into those who had never used e-cigarettes and those who had formerly used e-cigarettes, producing an eight-category exposure variable. Second, the principal multivariable model was additionally adjusted for asthma, COPD, diabetes, and cardiovascular disease. Comparisons were pre-specified against a single reference category rather than as pairwise contrasts among all groups, and no adjustment for multiple comparisons was applied; interpretation was therefore based on the magnitude and precision of effect estimates alongside the global Wald test rather than on threshold-crossing alone. A two-sided *p*-value below 0.05 was considered statistically significant. Analyses were conducted in StataNow 19.5 (StataCorp LLC, College Station, TX, USA).

## Results

### Characteristics of the study population

The analytic sample comprised 9274 adults, of whom 684 (weighted 7.4%) reported Long COVID. Participants with Long COVID differed significantly from those without on several characteristics (Table 1). Long COVID was more frequently reported by adults aged 35–64 years, women, and respondents living in the two most deprived SIMD quintiles (all *p* < 0.05). No significant difference was observed by ethnicity or educational attainment although ex-drinkers were somewhat overrepresented among those reporting Long COVID. The distribution of cigarette and e-cigarette use pattern differed overall by Long COVID status (global *p* = 0.003). Former cigarette smokers and exclusive e-cigarette users who were former smokers comprised larger weighted proportions among participants reporting Long COVID. Former e-cigarette users who had never smoked comprised 1.5% of participants with Long COVID and 2.4% of those without Long COVID.

**Table 1:** Characteristics of adults by survey-defined self-reported Long COVID status, Scottish Health Survey 2023–2024

Characteristic	No Long COVID *n* (%)	Long COVID *n* (%)	Total *n* (%)	*p*-value
*Age group, years*				<0.001
16–24	548 (10.6)	27 (6.9)	575 (6.2)	
25–34	1095 (15.8)	79 (12.9)	1174 (12.7)	
35–44	1130 (15.2)	123 (20.2)	1253 (13.5)	
45–54	1149 (16.0)	121 (19.7)	1270 (13.7)	
55–64	1566 (17.4)	171 (24.0)	1737 (18.7)	
65–74	1774 (13.8)	104 (10.6)	1878 (20.3)	
75+	1328 (11.1)	59 (5.6)	1387 (15.0)	
*Sex*				0.002
Male	3858 (48.2)	253 (40.1)	4111 (44.3)	
Female	4708 (51.5)	429 (59.6)	5137 (55.4)	
Prefer not to say	24 (0.3)	2 (0.4)	26 (0.3)	
*Ethnicity*				0.943
White Scottish	6347 (72.3)	502 (71.9)	6849 (73.9)	
Other White	1815 (20.5)	143 (20.5)	1958 (21.1)	
Minority ethnic	428 (7.2)	39 (7.6)	467 (5.0)	
*Area deprivation (SIMD)*				<0.001
Most deprived	1145 (18.1)	127 (24.0)	1272 (13.7)	
2nd	1496 (19.0)	141 (23.2)	1637 (17.7)	
3rd	2020 (20.1)	172 (21.7)	2192 (23.6)	
4th	2231 (21.7)	138 (15.9)	2369 (25.5)	
Least deprived	1698 (21.1)	106 (15.2)	1804 (19.5)	
*Educational attainment*				0.110
Degree or higher	3224 (38.4)	243 (35.9)	3467 (37.4)	
HNC/HND or equivalent	1034 (12.7)	102 (15.6)	1136 (12.2)	
Higher grade or equivalent	1208 (15.1)	98 (12.8)	1306 (14.1)	
Standard/other school level	2012 (22.5)	150 (22.0)	2162 (23.3)	
No qualifications	1112 (11.3)	91 (13.7)	1203 (13.0)	
*Alcohol use*				0.043
Never drank alcohol	735 (9.9)	56 (7.8)	791 (8.5)	
Ex-drinker	967 (9.8)	89 (13.9)	1056 (11.4)	
Drinks within guidelines	3875 (44.7)	309 (44.8)	4184 (45.1)	
Drinks beyond guidelines	2900 (34.0)	222 (31.9)	3122 (33.7)	
Missing/refused	113 (1.6)	8 (1.6)	121 (1.3)	
*Cigarette and e-cigarette use pattern*				0.003
Never cigarette/e-cigarette users	4577 (54.0)	323 (45.9)	4900 (52.8)	
Former cigarette smokers	2213 (22.6)	196 (26.9)	2409 (26.0)	
Former e-cigarette users, never smokers	144 (2.4)	8 (1.5)	152 (1.6)	
Excl. e-cig, former smoker	452 (5.5)	52 (8.4)	504 (5.4)	
Excl. e-cig, never smoker	108 (2.0)	11 (2.2)	119 (1.3)	
Excl. cigarette smokers	879 (10.6)	70 (11.1)	949 (10.2)	
Dual use	217 (3.0)	24 (4.0)	241 (2.6)	

Values are unweighted counts with survey-weighted column percentages for Long COVID status; total percentages are unweighted sample percentages. *p*-values are from design-based Pearson tests accounting for weighting, clustering, and stratification, except the product-use pattern *p*-value, which is from the global Wald test in the unadjusted survey-weighted logistic model

### Association between cigarette and e-cigarette use pattern and Long COVID

Results of the survey-weighted logistic regression analyses are presented in Table 2. Never cigarette/e-cigarette users served as the reference category. In the crude model, former cigarette smokers currently using neither product had higher odds of self-reported Long COVID (OR 1.40, 95% CI 1.12–1.75, *p* = 0.003) as did exclusive e-cigarette users who were former smokers (OR 1.81, 95% CI 1.28–2.54, *p* = 0.001). Former e-cigarette users who had never smoked showed no statistically detectable association (OR 0.72, 95% CI 0.32–1.62, *p* = 0.421). Estimates were also compatible with no difference for exclusive e-cigarette users who had never smoked (OR 1.33, 95% CI 0.63–2.79, *p* = 0.451), exclusive current cigarette smokers (OR 1.23, 95% CI 0.89–1.70, *p* = 0.210), and dual users (OR 1.58, 95% CI 0.96–2.59, *p* = 0.071). The overall unadjusted association was statistically significant (*F*(6610)=3.30, *p* = 0.0034).

**Table 2:** Association between cigarette and e-cigarette use pattern and survey-defined self-reported Long COVID among adults, Scottish Health Survey 2023–2024

Cigarette and e-cigarette use pattern	Crude OR (95% CI), p	Adjusted OR (95% CI), p	Adj. predicted probability % (95% CI)
Never cigarette/e-cigarette users	1.00 (reference)	1.00 (reference)	6.8 (5.9–7.8)
Former cigarette smokers	1.40 (1.12–1.75), 0.003	1.40 (1.12–1.76), 0.004	9.3 (7.8–10.8)
Former e-cigarette users, never smokers	0.72 (0.32–1.62), 0.421	0.89 (0.38–2.06), 0.782	6.1 (1.4–10.9)
Excl. e-cig, former smokers	1.81 (1.28–2.54), 0.001	1.56 (1.09–2.24), 0.014	10.2 (7.4–13.1)
Excl. e-cig, never smokers	1.33 (0.63–2.79), 0.451	1.44 (0.66–3.16), 0.361	9.5 (3.0–16.0)
Excl. cigarette smokers	1.23 (0.89–1.70), 0.210	0.99 (0.70–1.40), 0.955	6.8 (4.9–8.7)
Dual users	1.58 (0.96–2.59), 0.071	1.31 (0.77–2.22), 0.316	8.7 (4.9–12.5)

Odds ratios and adjusted predicted probabilities were estimated using survey-weighted logistic regression. The adjusted model included survey year, age group, sex, area deprivation, ethnicity, alcohol use, and educational attainment. The global adjusted Wald test for the seven-category product-use variable was *F*(6610) = 2.42, *p* = 0.0255

*OR* odds ratio, *aOR* adjusted odds ratio, *CI* confidence interval

After adjustment for age group, sex, area deprivation, ethnicity, alcohol use, educational attainment, and survey year, higher odds persisted among former cigarette smokers currently using neither product (aOR 1.40, 95% CI 1.12–1.76, *p* = 0.004) and exclusive e-cigarette users who were former smokers (aOR 1.56, 95% CI 1.09–2.24, *p* = 0.014). Former e-cigarette users who had never smoked showed no statistically detectable association (aOR 0.89, 95% CI 0.38–2.06, *p* = 0.782). Estimates were likewise compatible with no difference for exclusive e-cigarette users who had never smoked (aOR 1.44, 95% CI 0.66–3.16, *p* = 0.361), exclusive current cigarette smokers (aOR 0.99, 95% CI 0.70–1.40, *p* = 0.955), and dual users (aOR 1.31, 95% CI 0.77–2.22, *p* = 0.316). Adjusted predicted probabilities were 6.8% (95% CI 5.9–7.8) among never cigarette/e-cigarette users, 9.3% (7.8–10.8) among former cigarette smokers, and 10.2% (7.4–13.1) among exclusive e-cigarette users who were former smokers. The overall adjusted association remained statistically significant (*F*(6610) = 2.42, *p* = 0.0255).

In the eight-category sensitivity analysis, former smokers who had never used e-cigarettes (*n* = 2087; aOR 1.41, 95% CI 1.11–1.78) and former smokers who had formerly used e-cigarettes (*n* = 322; aOR 1.38, 95% CI 0.87–2.20) had closely similar estimates, and their coefficients did not differ (*p* = 0.948). In the sensitivity model additionally adjusted for asthma, COPD, diabetes, and cardiovascular disease (*n* = 9266), the associations remained for former cigarette smokers (aOR 1.35, 95% CI 1.08–1.70, *p* = 0.010) and exclusive e-cigarette users who were former smokers (aOR 1.48, 95% CI 1.03–2.12, *p* = 0.033); the global product-use test remained significant (*p* = 0.0405). Full results are provided in Supplementary Tables S1 and S2.

## Discussion

In this nationally representative cross-sectional analysis of 9274 Scottish adults, higher adjusted odds of survey-defined self-reported Long COVID were observed among former cigarette smokers currently using neither product and among former smokers currently using e-cigarettes exclusively. Former e-cigarette users who had never smoked showed no elevation, while current e-cigarette users who had never smoked, exclusive current cigarette smokers, and dual users had statistically imprecise estimates compatible with no difference from never cigarette/e-cigarette users. The overall product-use variable remained associated with Long COVID after covariate adjustment, and the two elevated associations persisted after additional adjustment for asthma, COPD, diabetes, and cardiovascular disease. Nevertheless, the cross-sectional pattern cannot isolate an independent effect of current e-cigarette use.

Our findings are broadly consistent with the most directly comparable study, a BRFSS analysis that found no independent association between current or former e-cigarette use and post-acute COVID-19 syndrome after adjustment [17]. The main contribution of the present study is the separation of both current and former e-cigarette users by cigarette-smoking history. Former e-cigarette users who had never smoked did not show elevated odds, and the estimate among current e-cigarette users who had never smoked was imprecise. By contrast, elevated odds were observed in two groups of former smokers who no longer smoked cigarettes: those not using product and those using e-cigarettes exclusively. However, because exclusive current smokers and dual users also had cigarette-smoking histories but did not show elevated adjusted odds, the findings should not be interpreted as a simple effect of any prior smoking history. Pooling all current e-cigarette users into one category may obscure this heterogeneity.

Several explanations for the elevated odds among former smokers warrant consideration. Cigarette and e-cigarette use were measured at the survey interview, after the COVID-19 infection and symptom course that defined the outcome, so the observed product-use categories may partly represent behavioral responses to illness. People who experienced severe acute disease or persistent symptoms may have stopped smoking, switched to e-cigarettes, or reduced their product use, enriching the former-smoker groups with individuals at higher underlying risk. Some former smokers may also have quit earlier because of smoking-related diseases that are themselves associated with Long COVID. Additional adjustment for asthma, COPD, diabetes, and cardiovascular disease produced only modest attenuation, but it cannot remove confounding by unmeasured disease burden, infection severity, healthcare contact, or the timing of smoking cessation and switching. Reverse causation and health-related selection therefore remain central explanations.

The null finding for exclusive current cigarette smoking is one of the most difficult aspects of these results to interpret. Some population-based studies have reported positive associations between current smoking and Long COVID [6, 19], but prospective evidence has been less consistent. A recent UK cohort analysis by Adebisi and colleagues found that the apparent association between smoking and Long COVID was attenuated after adjustment and appeared to operate partly through pre-existing long-term illness [7]. Our findings are broadly compatible with that interpretation. At the same time, current smokers who survived acute COVID-19, remained sufficiently well to participate in a household survey, and continued to identify as smokers may constitute a selected subgroup. The null estimate should therefore not be interpreted as evidence that cigarette smoking is protective or harmless. Similar selection and survivorship processes may also affect estimates in the other product-use groups.

Overall, the pattern of results does not support a single mechanistic explanation. A model in which current cigarette smoking alone determines Long COVID risk is difficult to reconcile with the null estimate for current smokers, while the available data are insufficient to establish or exclude an independent contribution of current e-cigarette use. The most defensible interpretation is that the findings reflect a combination of previous cigarette exposure, health-related cessation or switching, reverse causation, residual confounding, and selection processes that cannot be disentangled in a cross-sectional survey.

This study has several strengths. It used a large, nationally representative sample of adults in Scotland and accounted for the complex survey design. It also classified cigarette and e-cigarette use in greater detail than many previous studies by separating current and former e-cigarette users according to cigarette-smoking history and distinguishing exclusive cigarette smoking from dual use. The seven-category classification removed never-smoking former e-cigarette users from the former-smoker group, and the findings were examined in sensitivity analyses using a finer eight-category classification and additional adjustment for selected comorbidities.

Several limitations should also be acknowledged. Most importantly, the cross-sectional design precludes causal inference and does not establish whether cigarette smoking cessation, e-cigarette initiation, switching, or dual use preceded SARS-CoV-2 infection or the onset of persistent symptoms. Exposure status measured at interview may therefore be a consequence rather than a cause of illness. People with more severe acute COVID-19 or persistent symptoms may have been particularly likely to stop smoking or switch products, while former smokers may have quit because of pre-existing smoking-related disease. The product-use categories should consequently not be interpreted as pre-infection exposure states.

Both product use and Long COVID were self-reported and may have been misclassified. The survey classified Long COVID using symptoms continuing for more than four weeks after first having COVID-19, a broader threshold than the WHO clinical definition, which generally places onset within three months and requires symptoms to last for at least two months, and the 2024 National Academies definition, which requires an infection-associated chronic condition to be present for at least three months [21, 22]. The survey outcome may therefore include shorter-duration post-acute symptoms that would not meet these definitions. The former e-cigarette user, never-smoker group, current e-cigarette user, never-smoker group, and dual-user group were small, producing wide confidence intervals.

Detailed product-use histories were incompletely measured. Smoking duration and time since cessation were available only for subsets of former smokers, while current cigarette intensity was available only for current smokers. Daily versus non-daily e-cigarette use was available for a subset of current users, but e-cigarette duration was unavailable. Consequently, cumulative cigarette exposure and detailed product-use intensity could not be incorporated consistently across all exposure groups. COVID-19 vaccination status and acute COVID-19 severity were not available in the pooled analytic file, and BMI was unavailable for approximately one quarter of participants. Although selected comorbidities were included in a sensitivity analysis, residual confounding by other health conditions and infection-related factors remains possible. The findings should therefore be interpreted as descriptive associations rather than causal effects.

## Conclusions

In this pooled analysis of the 2023 and 2024 Scottish Health Survey, 7.4% of adults reported survey-defined Long COVID. Compared with adults who had never smoked cigarettes or used e-cigarettes, higher adjusted odds were observed among former cigarette smokers currently using neither product and among former smokers currently using e-cigarettes exclusively. No statistically detectable association was observed among former e-cigarette users who had never smoked, current e-cigarette users who had never smoked, exclusive cigarette smokers, or dual users, although several estimates were imprecise. The findings persisted after additional adjustment for selected comorbidities but should not be interpreted causally because smoking cessation or switching may have occurred after COVID-19 infection or Long COVID onset. Longitudinal studies with pre-infection exposure measurement, cumulative smoking histories, product-use intensity, vaccination status, and acute disease severity are required before firmer conclusions can be drawn.

## Supplementary Information

Below is the link to the electronic supplementary material.

Supplementary file1 (DOCX 30 KB)

## Data Availability

The data underlying this article are available from the UK Data Service. The Scottish Health Survey data used in this study were accessed under license and therefore cannot be shared directly by the authors. Interested researchers may obtain the data from the UK Data Service, subject to the required registration and access procedures.
