# Supplementary material for: Cigarette smoking history, e-cigarette use, and Long COVID: a population-based analysis
Source: Intern Emerg Med. 2026 Aug 28;21(8):2985–92. doi: 10.1007/s11739-026-04512-w (PMC13751570; doi:10.1007/s11739-026-04512-w)
Supplement: Supplementary file 1 [file 11739_2026_4512_MOESM1_ESM.docx]

**Supplementary material**

**Cigarette smoking history, e-cigarette use, and Long COVID: a population-based analysis**

Yusuff Adebayo Adebisi and Anas Ali Alhur

**Supplementary methods**

Two survey-weighted sensitivity analyses were conducted. First, the seven-category cigarette and e-cigarette use variable was expanded to eight categories by separating former cigarette smokers currently using neither product into those who had never used e-cigarettes and those who had formerly used e-cigarettes. A post-estimation Wald test compared the two former-smoker coefficients. Second, the primary multivariable model was additionally adjusted for doctor-diagnosed asthma, chronic obstructive pulmonary disease, diabetes excluding gestational diabetes, and cardiovascular disease excluding diabetes and hypertension. The primary covariates were survey year, age group, sex, Scottish Index of Multiple Deprivation quintile, ethnicity, alcohol use, and educational attainment. All models incorporated the pooled survey weights, strata, and primary sampling units.

**Supplementary Table S1. Eight-category sensitivity analysis of cigarette and e-cigarette use pattern and survey-defined self-reported Long COVID**

| **Product-use category** | **N** | **Long COVID cases** | **Crude OR (95% CI), p** | **Adjusted OR (95% CI), p** | **Adjusted probability, % (95% CI)** |
| --- | --- | --- | --- | --- | --- |
| Never cigarette/e-cigarette users | 4,900 | 323 | 1.00 (reference) | 1.00 (reference) | 6.8 (5.9–7.8) |
| Former smoker, never e-cigarette user | 2,087 | 164 | 1.39 (1.10–1.74), 0.005 | 1.41 (1.11–1.78), 0.005 | 9.3 (7.7–11.0) |
| Former smoker, former e-cigarette user | 322 | 32 | 1.49 (0.94–2.34), 0.087 | 1.38 (0.87–2.20), 0.169 | 9.2 (5.7–12.7) |
| Former e-cigarette user, never smoker | 152 | 8 | 0.72 (0.32–1.62), 0.421 | 0.89 (0.38–2.06), 0.781 | 6.1 (1.4–10.8) |
| Exclusive e-cigarette user, former smoker | 504 | 52 | 1.81 (1.28–2.54), 0.001 | 1.56 (1.09–2.24), 0.014 | 10.2 (7.4–13.1) |
| Exclusive e-cigarette user, never smoker | 119 | 11 | 1.33 (0.63–2.79), 0.451 | 1.44 (0.66–3.16), 0.362 | 9.5 (3.0–16.0) |
| Exclusive cigarette smoker | 949 | 70 | 1.23 (0.89–1.70), 0.210 | 0.99 (0.70–1.40), 0.955 | 6.8 (4.9–8.7) |
| Dual user | 241 | 24 | 1.58 (0.96–2.59), 0.071 | 1.31 (0.77–2.22), 0.316 | 8.7 (4.9–12.5) |

The adjusted model included survey year, age group, sex, area deprivation, ethnicity, alcohol use, and educational attainment. The former-smoker coefficients for those who had never used e-cigarettes and those who had formerly used e-cigarettes did not differ (adjusted Wald F(1,615)=0.00, p=0.948). The global adjusted Wald test for the eight-category variable was F(7,609)=2.09, p=0.0426. OR, odds ratio; CI, confidence interval. Estimates for the categories not affected by the split differ from Table 2 only in the final reported digit because the model was refitted with an additional category.

**Supplementary Table S2. Sensitivity analysis additionally adjusted for selected comorbidities**

| **Product-use category** | **Primary adjusted OR (95% CI), p** | **Comorbidity-adjusted OR (95% CI), p** | **Comorbidity-adjusted probability, % (95% CI)** |
| --- | --- | --- | --- |
| Never cigarette/e-cigarette users | 1.00 (reference) | 1.00 (reference) | 7.0 (6.0–8.0) |
| Former cigarette smokers | 1.40 (1.12–1.76), 0.004 | 1.35 (1.08–1.70), 0.010 | 9.2 (7.7–10.6) |
| Former e-cigarette users, never smokers | 0.89 (0.38–2.06), 0.782 | 0.89 (0.39–2.05), 0.783 | 6.3 (1.5–11.0) |
| Exclusive e-cigarette users, former smokers | 1.56 (1.09–2.24), 0.014 | 1.48 (1.03–2.12), 0.033 | 9.9 (7.2–12.7) |
| Exclusive e-cigarette users, never smokers | 1.44 (0.66–3.16), 0.361 | 1.40 (0.63–3.10), 0.413 | 9.4 (2.9–15.9) |
| Exclusive cigarette smokers | 0.99 (0.70–1.40), 0.955 | 0.91 (0.63–1.30), 0.588 | 6.4 (4.5–8.2) |
| Dual users | 1.31 (0.77–2.22), 0.316 | 1.15 (0.68–1.95), 0.609 | 7.9 (4.5–11.4) |

The comorbidity-adjusted model included the primary covariates plus doctor-diagnosed asthma, COPD, diabetes excluding gestational diabetes, and cardiovascular disease excluding diabetes and hypertension. The sensitivity-analysis sample included 9,266 participants because eight participants had non-substantive responses for at least one comorbidity. The global adjusted Wald test for product-use pattern was F(6,610)=2.21, p=0.0405. OR, odds ratio; CI, confidence interval; COPD, chronic obstructive pulmonary disease.
